# Azelaic acid reduces arsenic-induced toxicity and inflammation in the rat islets of langerhans

**DOI:** 10.22038/ijbms.2025.84651.18311

**Published:** 2025

**Authors:** Sara Mostafalou, Fatemeh Moafi-Madani, Maryam Baeeri, Mahban Rahimifard, Hamed Haghi-Aminjan

**Affiliations:** 1 Department of Pharmacology and Toxicology, School of Pharmacy, Ardabil University of Medical Sciences, Ardabil, Iran; 2 Toxicology and Diseases Group, Pharmaceutical Sciences Research Center, Tehran University of Medical Sciences, Tehran, Iran; 3 Pharmaceutical Sciences Research Center, Ardabil University of Medical Sciences, Ardabil, Iran

**Keywords:** Apoptosis, Azelaic acid, Inflammation, Insulin, Oxidative stress, Sodium arsenite

## Abstract

**Objective(s):**

Arsenic is classified as a toxic metal that is naturally found in the Earth’s crust, and long-term exposure to it can result in chronic human disorders like cancer and diabetes. Azelaic acid (AZA), a natural dicarboxylic acid, has been reported to have anti-oxidant and anti-inflammatory effects; hence, it may protect against the metabolic toxicity of arsenic. This study aimed to investigate whether AZA could ameliorate sodium arsenite (SA) toxicity toward rat islets of Langerhans.

**Materials and Methods:**

Pancreatic Islets of Langerhans isolated from adult male Wistar rats were divided into four groups of 10: control, SA, AZA, and SA plus AZA. Twenty-four hours after incubation, cell viability, cell death pathways, reactive oxygen species (ROS), inflammatory factor gene expression, and insulin secretion were evaluated.

**Results:**

SA dose-dependently decreased cell viability, increased apoptosis, ROS generation, expression of inflammatory mediators (NF-κB, IL-1β, and TNF-α), and insulin secretion. AZA was able to ameliorate all these changes significantly.

**Conclusion:**

Our results indicate that SA can potentially disrupt cellular homeostasis and function in the islets of Langerhans and can increase the risk of metabolic diseases such as diabetes. On the other hand, AZA protected islets of Langerhans against the toxic effects of SA, seemingly due to its anti-apoptotic, anti-inflammatory, and anti-oxidant properties, indicating that AZA may have the potential to run intracellular mechanisms beneficial for coping with the metabolic toxicity of arsenic.

## Introduction

Arsenic is a natural metalloid found in water, soil, and air, and is one of the most toxic pollutants of drinking water in the world. Epidemiological studies have evidenced a positive association between exposure to arsenic and a higher incidence of human endocrine and metabolic disorders, especially diabetes (1). Although genetic and lifestyle factors are considered the leading causes of metabolic disorders, exposure to environmental toxicants, particularly heavy metals, has been shown to increase the risk of diabetes (2-4). The link of arsenic with diabetes has also been confirmed by experimental studies evaluating the toxic effect of arsenic on pancreatic islets of Langerhans and insulin-responsive tissues like the liver, muscles, and adipose tissues. Arsenic has been shown to cause glucose intolerance by disrupting secretion of insulin from pancreatic β-cells (5). Although different modes of action have been suggested for the toxic effects of arsenic, research has shown that uncontrolled circulation of intracellular reactive molecules such as reactive oxygen species (ROS) and consequent oxidative damage to macromolecules are mainly involved in arsenic-induced disturbance in cellular homeostasis. Oxidative stress along with provoking inflammatory responses can activate mitochondrial dependent apoptotic or necrotic signals leading to depletion of cellular energy reservoirs and cell death (6, 7). 

Azelaic acid (AZA), produced by *Malassezia furfur*, is a naturally occurring dicarboxylic acid found in rye, barley, cereals, and animals. It is a complex molecule with diverse physiological activities, including anti-inflammatory, antibacterial, keratolytic, comedolytic, and anti-oxidant effects. AZA has been shown to be anti-inflammatory by eliminating oxygen-free radicals (8). Further, one study has reported that AZA has a protective effect against diabetic complications induced by oxidative stress of a high-fat diet in mice (9). The present work was conducted to study the toxicity of sodium arsenite (SA) in the pancreatic islets by evaluating insulin secretion, markers of oxidative stress, expression of pro-inflammatory factors and apoptotic potential and to determine whether AZA was able to counteract the toxic effects of SA in the islets of Langerhans isolated from the pancreas of adult male Wistar rats.

## Materials and Methods

### Chemicals

ROS ELISA kit and Apo flowEx FITC Kit were respectively procured from Zellbio GmbH (Germany) and the Exbio Company (Czech Republic). RNA extraction kit and cDNA synthesis kit were obtained from BioFACT Company (Korea). SYBR®Premix Ex Taq was obtained from Takara Bio Inc. The specific primers for evaluating mRNA expression were prepared from Eurofins genomics (Germany). Ketamine/Xylazine, sodium arsenit, azelaeic acid, EDTA, HEPES, dithiothreitol (DTT), phosphate buffered saline (PBS), DEPC-treated water, fetal bovin serum (FBS), dimethyl sulfoxide (DMSO), Krebs-ringer buffer, and reactive buffers were obtained from Sigma Aldrich Co. 

### Animals: supplier

Male Wistar rats were obtained from the animal house of the Pasteur Institute in Tehran (Iran). In this study, a total of 20 adults male Wistar rats (age 8 weeks, weight 200–250 g) were accommodated in standard cages (26.6×45.5×18 cm) (4 rats in each cage) with 23±1 ℃ temperature and a 12 hr light/12 hr dark cycle. Free access to water and food were provided for all rats throughout the study. All experiments were conducted between 8:00 and 14:00 during the light cycle. The study was approved by the ethics committee at Ardabil University of Medical Sciences (IR.ARUMS.AEC.1401.028).

### Isolation of islets of langerhans

To prepare and isolate the islets of Langerhans, first, the rats were anesthetized using 90 mg of ketamine and 10 mg of xylazine. After complete anesthesia, a midline incision was made, and the main duct of the pancreas was closed at the junction of the intestine and then cannulated. The channel was perfused with 15 cc of Krebs buffer so that pancreatic tissues were swallowed. The pancreas was cut and placed inside a tube containing Krebs buffer on the ice chamber. Then, it was cut into small pieces with autoclaved scissors under the hood and centrifuged at 1700 g and 4 °C for one minute. After adding the collagenase enzyme to the precipitate, it was shaken in a 37 °C water bath for 3–5 min. Collagenase-induced digestion was stopped with BSA, and then the sediments in the tubes were washed twice with Krebs buffer. The contents of the tubes were transferred to plates, and islets of approximately the same size were picked up using a sampler under a stereo microscope. Isolated islets of Langerhans were incubated for 24 hr and then divided into groups of 10 each. The groups of islets were treated with the study materials at 37 °C for 24 hr (10).

### Treatment of pancreatic islets

In order to determine the median lethal concentration (LC50) of SA, groups of 10 islets of Langerhans were treated with logarithmic concentrations of SA (1, 5, 10, 50, 100, 500, and 1000 μM). Then, the islets of Langerhans were treated with a combination of SA (LC50) and the logarithmic concentrations of AZA (1, 10, 50, 100, 500, 1000, and 2000 μM) to find the median effective concentration (EC50) of AZA. 

### Assessment of cell viability

After 24 hr, we removed the culture medium, added 50 µl of MTT solution with a concentration of 5 mg/ml in phosphate buffer to each well, and placed them in the incubator for four hours. Then 150 µl DMSO was added to each well and left for 20 min inside the shaker incubator. Finally, 200 µl of the islet medium was extracted and transferred to a 96-well plate, put in the microtiter plate reader, and its absorbance was read at 570 nm (11). 

### Assessment of ROS formation

The islets of Langerhans were separated, homogenized with extraction buffer, and centrifuged at 3864 g for 5 min. Then 25 μl of the supernatant was added to a mix of assay buffer (80 μl) and DCFH-DA (10 μl). The solution was incubated at 37 °C for 15 min, and the absorbance was read every 5 to 60 min using a microplate reader equipped with the fluorescence analyzer. ROS level was calculated based on a standard curve and reported as units per mg of tissue protein (12).

### Assessment of insulin secretion

After 24 hr of treatment, we added 1 mL Krebs medium to the islets and centrifuged them at 3000 g for 1 min. We removed the supernatant and incubated the islets of Langerhans in a medium containing 2.8 mM glucose for 30 min. In the next step, the tubes of islets were divided into two groups. One group of islets (basal phase) was incubated with 2.8 mM glucose, while the other group (stimulant phase) was treated with 16.7 mM glucose. After one hour, the tubes were centrifuged, and the supernatants were used to evaluate insulin secretion. Insulin content was measured using an insulin ELISA kit, according to the manufacturer’s instructions. The results were reported in μg per mg protein. The protein content of the samples was assessed using the Bradford reagent according to a protocol previously described (10). 

### Apoptosis/necrosis test

Flow cytometry-based methodologies are commonly used to study cellular death pathways, including apoptosis and necrosis. Propidium iodide (PI) plus annexin V is a flow cytometry dye indicating a cell is alive, apoptotic, or necrotic according to the changes in the permeability and stability of the cellular plasma membrane. Cell viability was studied using the ApoFlowEx apoptosis detection kit in this study. According to the manufacturer’s instructions, cells were treated with fluorescein isothiocyanate (FITC)-conjugated PI and annexin V and incubated at room temperature for five minutes. Then, the cells were tested by flow cytometry using the German-made Cyflow Space-Partec device. 

### Investigation of gene expression changes by real-time PCR

To evaluate changes in the gene expression of NF-kB, IL-1β, and TNF-α, mRNA extraction, cDNA synthesis, and real-time PCR were performed subsequently. First, RNA of Langerhans Islands samples was extracted by homogenizing tissues with a BioFACT kit solution. A Nano-Drop UV–vis Spectro-photometer (Thermo Fisher Scientific, CA) was used to determine the concentration of RNA. Transcription of 1 μg/μl mRNA and synthesis of cDNA was done using the BioFACT cDNA synthesis kit. The specific primers were prepared from Eurofins genomics (Eurofins genomics, Germany). For real-time PCR reaction, we used the SYBR green master mix. A Light Cycler 96 (Roche Applied Sciences, USA) was used to achieve the cycle number of each reaction. The values were normalized to GAPDH mRNA, and the relative expression of each mRNA was represented as 2^−ΔΔCt^. The primers with the following sequences were used in this study (13, 14):

IL-1β reverse: GTCCTTAGCCACTCCTTCTG 

IL-1β forward: AGCCAGAGTCATTCAGAGCAA 

TNF-α reverse: TCCACTCAGGCATCGACATT 

TNF-α forward: ACACACGAGACGCTGAAGTA 

NF-kB reverse: AGGTATGGGCCATCTGTTGA 

NF-kB forward: TTCAACATGGCAGACGACGA 

GAPDH reverse: AAGACGCCAGTAGACTCCAC

GAPDH forward: GTATGACTCTACCCACGGCA

### Data analysis

Data were analyzed using the one-way analysis of variance (ANOVA) test and Tukey’s multiple comparisons (*post hoc*) test. In this way, the results of 5 tests were expressed in terms of mean and standard error (Mean ± SEM), and results with a *P*-value lower than 0.05 were considered statistically significant.

## Results

### Cell viability

**Figure 1:**
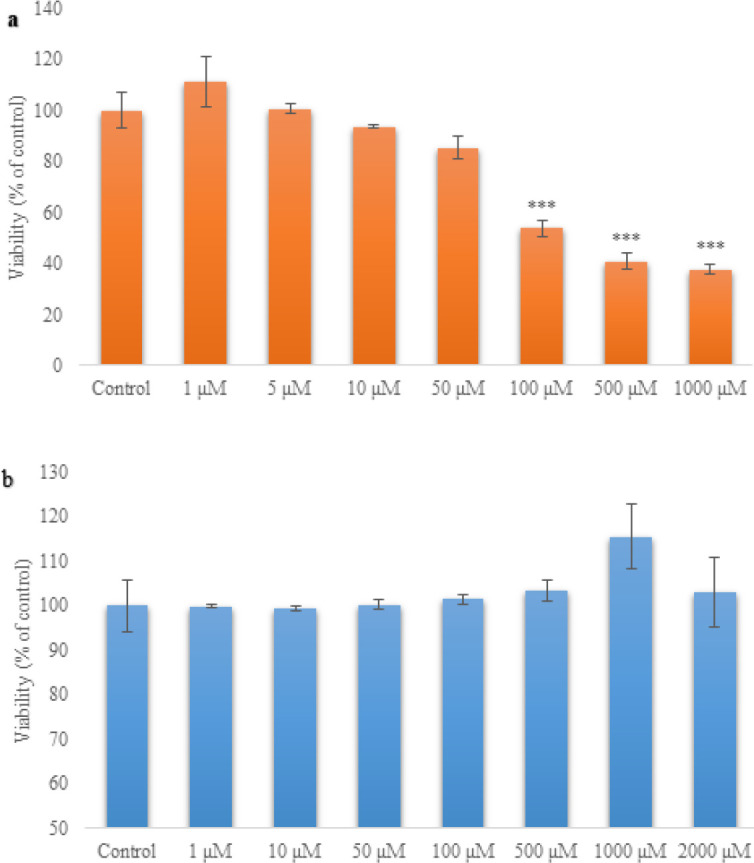
Effect of different concentrations of SA (1, 5, 10, 50, 100, 500, and 1000 µM) (A) and AZA (1, 10, 50, 100, 500, 1000, and 2000 µM) on the viability of rat pancreatic islets

Treatment of pancreatic islets with logarithmic concentrations of SA ranging from 1-1000 μM for 24 hr showed a dose-dependent lowering effect in the viability of pancreatic islets. In this experimental setup, 100 μM was established as the LC50 of SA. To establish the EC50 of AZA, a range of concentrations (1–2000 μM) was used against SA (LC50), and 1000 μM was found to have the most protective effect against SA toxicity (Figure 1). 

### ROS Formation

**Figure 2:**
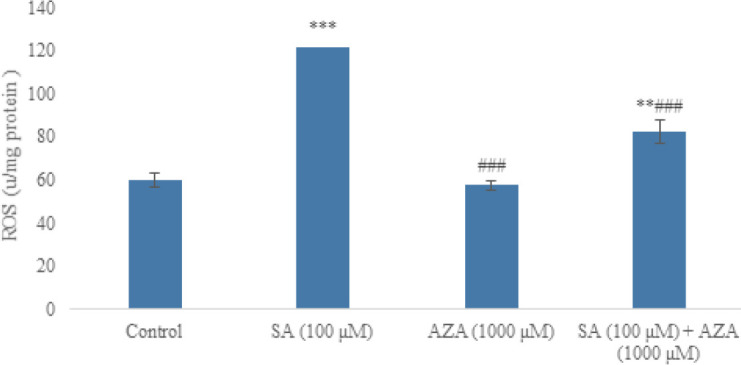
Effect of SA (100 µM) and AZA (1000 µM) alone and in combination on the ROS formation in rat pancreatic islets

As shown in Figure 2, SA significantly increased the level of ROS in the pancreatic islets (*P*<0.001). There was no difference between the control and AZA groups in terms of ROS formation. A significant decrease (*P*<0.001) was found in the ROS level of islets treated with SA + AZA in comparison with the SA group (Figure 2).

### Apoptosis vs. necrosis

**Figure 3:**
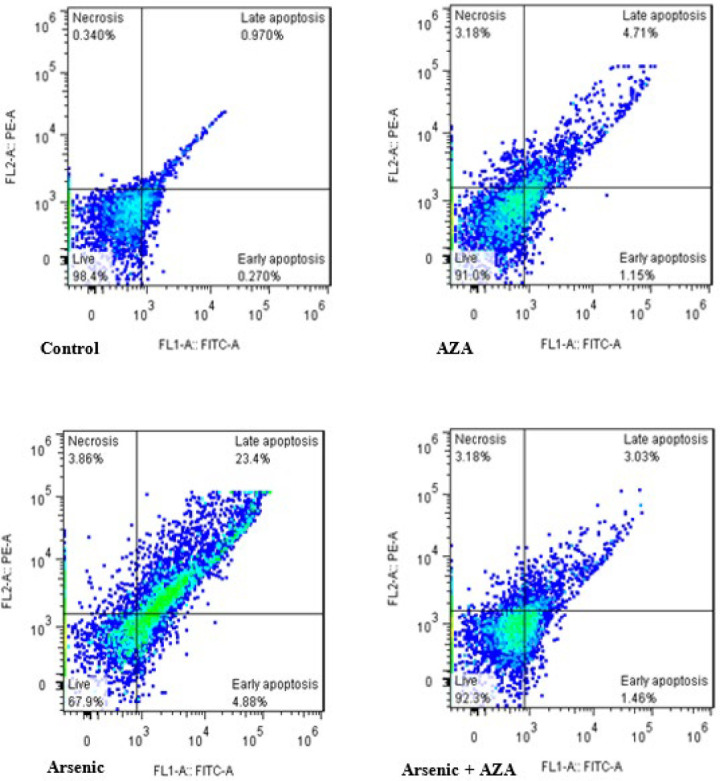
Flow cytometry assessment of cell death pathways in the rat pancreatic islets under the effect of SA and AZA, alone and in combination

As shown in Figure 3, the percentages of live cells in the control and AZA groups were 98.4% and 92.3%, respectively, while this percentage decreased to 67.9% in the islets treated with SA. Co-treatment of islets with SA + AZA resulted in 91% viability. SA caused an increase in the number of apoptotic cells up to 28%, while co-treatment with AZA decreased apoptotic cells to 5.86%. The number of necrotic cells in all groups was higher than control and nearly in the same range (3%) (Figure 3). 

### Gene expression changes assessed by real-time PCR

**Figure 4:**
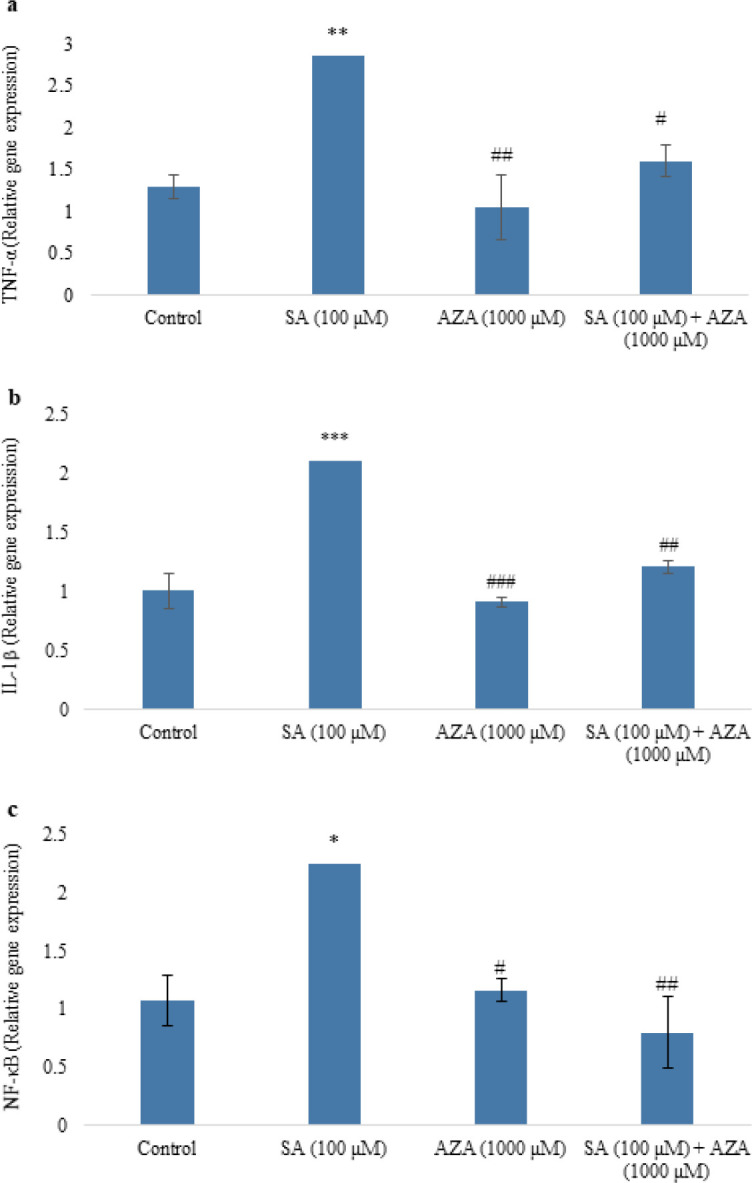
Effect of SA (100 µM) and AZA (1000 µM), alone and in combination, on mRNA expression of TNF-α (A), IL-1β (B), and NF-κB (C) in the rat pancreatic islets

As shown in Figure 4A, SA significantly (*P*<0.01) increased the expression of TNF-α in the pancreatic islets, while the AZA-treated islets didn’t have a significant difference with the control. The expression of TNF-α in the islets co-treated with SA and AZA was lower (*P*<0.05) than that of the SA group (Figure 4a). The level of IL-1 gene expression in the islets was significantly (*P*<0.001) increased after 24 hr treatment with SA. In comparison with control, AZA did not have any significant effect on the expression of IL-1. At the same time, co-treatment of islets with SA and AZA caused a marked (*P*<0.01) decrease in the expression of IL-1 compared with SA-treated islets (Figure 4b). SA also increased the expression of the NF-κB gene (*P*<0.05), but AZA alone had no significant effect in the islets in comparison with the control. Expression of NF-κB gene in co-treated islets with SA and AZA was significantly lower (*P*<0.01) than that of islets treated with SA (Figure 4c).

### Insulin secretion

**Figure 5:**
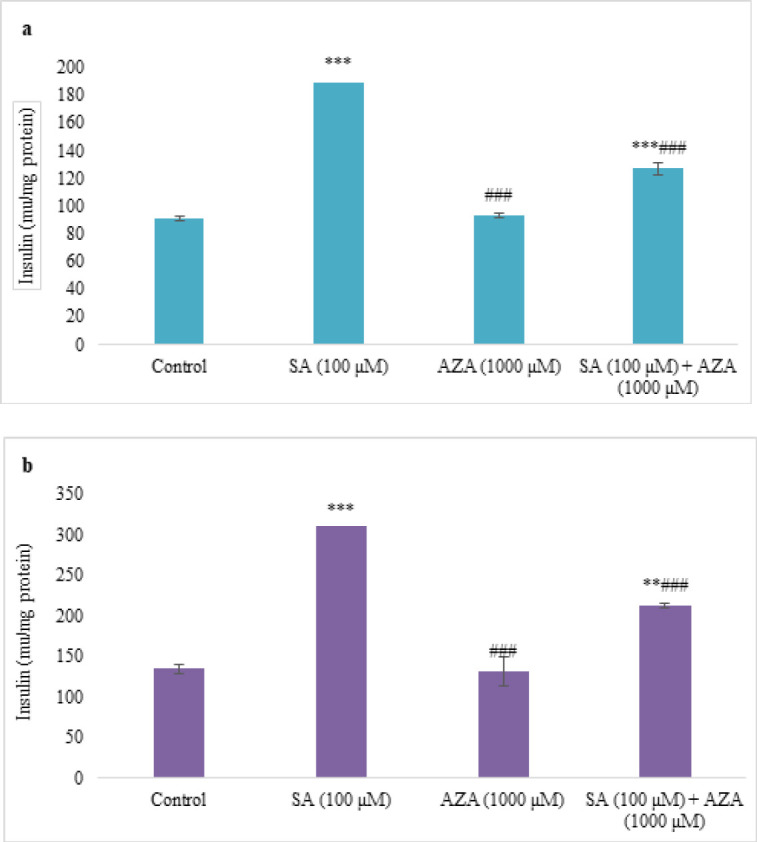
Effect of SA (100 µM) and AZA (1000 µM) alone and in combination on insulin secretion from rat pancreatic islets in basal (A) and glucose-stimulated (B) phases

As indicated in Figure 5, SA dramatically (*P*<0.001) increased insulin secretion at both basal and stimulated phases, while AZA did not change it. There was also a significant decrease (*P*<0.001) in insulin secretion of islets treated with SA + AZA in comparison with the SA group (Figure 5).

## Discussion

In this study, we investigated the toxicity of SA in the pancreatic islets of Langerhans obtained from male Wistar rats and the ability of AZA to protect against its harmful effects. At first, cell viability was evaluated with MTT assessment, based on which the LC50 of SA and EC50 of AZA were obtained by dose-response relationship. The results indicated that SA decreased the viability of pancreatic islets, while the addition of AZA prevented cell damage caused by SA. 

The survival of pancreatic islets was also evaluated using the flow cytometric method to reveal detailed effects of SA and AZA, alone and in combination, on the apoptotic or necrotic states of the cells. As indicated, SA decreased the percentage of live cells by 30%, and the primary mechanism of this increase in cellular death was shown to be apoptosis rather than necrosis. This result confirms previous reports on the pro-apoptotic effect of arsenic in pancreatic cells. In a study conducted on pancreatic β-cells, arsenic was shown to significantly increase the phosphorylation of JNK, ERK, and p38 MAPK along with increasing ROS generation, and these results indicate that activation of MAPKs may be involved in the apoptosis induced by SA (15). Increased ROS production through mitochondrial pathways (cytochrome c release) and consequent activation of MAPKs and expression of pro-inflammatory cytokines have been shown to activate caspase 3 and lead to apoptotic events (16). In our study, adding AZA to the islets treated with SA recovered cell viability and reduced the number of apoptotic cells close to the control group. AZA has already been shown to have anti-oxidant properties and can prevent deleterious effects of ROS and oxidative damage (17, 18). AZA has probably prevented SA-induced apoptotic cell death in the pancreatic islets by counteracting ROS effects and MAPK pathways. Our results indicate that AZA prevented SA-induced ROS generation in the pancreatic islets. This observation was per the findings of cellular viability and apoptotic cell death under the effects of SA and AZA. 

Increased production of ROS in the cells can create an imbalanced state of oxidant/anti-oxidant ratio, leading to oxidative damage to the cellular macromolecules, affecting their structure and functions. Such a situation can increase consecutive phosphorylation reactions in MAPK and JNK pathways, leading to activation of the transcription factor AP- 1 and NF-κB, which can result in up-regulation of pro-inflammatory cytokines such as TNF-α, IL-1β, and IL-6 (16, 19). 

An *in vitro* study conducted on normal keratinocytes investigated the effect of AZA on inflammatory reaction to ultraviolet-B (UVB) light. The results indicated that AZA could suppress UVB light-induced expression of pro-inflammatory cytokines, including IL-1β, IL-6, and TNF-α, translocation of NF-κB, phosphorylation of p38 mitogen-activated protein kinase and stress-activated protein kinase. Furthermore, AZA induced the expression of peroxisome proliferators-activated receptor-γ (PPAR-γ), a nuclear receptor activated by fatty acids and lipid peroxidation products. PPAR-γ is crucial in controlling inflammation and oxidative stress (18). 

In our study, SA could increase the expression of NF-κB and pro-inflammatory cytokines, including TNF-α and IL-1β in the pancreatic islets. As expected, co-administration of AZA significantly reduced the expression of NF-κB, TNF-α, and IL-1β, which support AZA’s anti-oxidant and anti-inflammatory properties. In a study conducted on C57BL/6J mice with type 2 diabetes induced by a high-fat diet, administration of AZA was able to normalize the level of inflammatory cytokines, lipids, and adipokines. AZA treatment also increased the expression of genes involved in scavenging ROS and insulin signaling pathways like IRS -1, PI3K, and Akt, leading to increased insulin sensitivity (20).

In our study, the amount of insulin secretion from the islets of Langerhans treated with SA increased when exposed to basal and stimulating glucose concentrations. This effect is not in parallel with previous findings on the decreasing effect of low-dose arsenic on glucose-stimulated insulin secretion (GSIS) in the islets of Langerhans. The results of previous studies suggested that arsenic at low levels can provoke an adaptive response against oxidative stress, leading to increased anti-oxidant capacity, which can impair ROS-mediated metabolic signals for GSIS and consequently decrease GSIS (21-24). These contradictive effects in our study can be explained by increased apoptotic cell death induced by SA, so the increased concentration of insulin in the media of SA-treated pancreatic islets may be related to the increased release of cellular contents due to apoptosis. There can be other explanations for this contradiction, such as differences in the system model used, the dose and duration of SA exposure, and the time periods used for measuring GSIS. Subsequently, the addition of AZA to the pancreatic islets in our study could decrease SA-induced insulin secretion in both basal and glucose-stimulated phases. This observation can be interpreted as AZA’s anti-oxidant, anti-inflammatory, and anti-apoptotic properties. Insulin concentration in the media of pancreatic islets in our study was negatively associated with cell survival.

The tests were repeated four times, and the results were expressed as Mean ± SEM. SA: sodium arsenite, AZA: azelaic acid

*** Significantly different from the control group at* P*-value<0.001.

The tests were repeated four times, and the results were expressed as Mean ± SEM. SA: sodium arsenite, AZA: azelaic acid; ROS: Reactive oxygen species

*** Significantly different from the control group at *P*-value<0.001.

** Significantly different from the control group at *P*-value<0.01.

### Significantly different from the SA group at *P*-value<0.001.

The lower left square indicates the number of living cells, the lower right square is the number of early apoptotic cells, the upper right square is the number of late apoptotic cells, and the upper left square is the number of necrotic cells.

SA: sodium arsenite, AZA: azelaic acid

The tests were repeated four times, and the results were expressed as Mean ± SEM. SA: sodium arsenite, AZA: azelaic acid

* Significantly different from the control group at *P*-value<0.05.

** Significantly different from the control group at *P*-value<0.01.

*** Significantly different from the control group at *P*-value<0.001.

# Significantly different from the SA group at *P*-value<0.05.

## Significantly different from SA group at *P*-value<0.01.

### Significantly different from the SA group at *P*-value<0.001.

The tests were repeated four times, and the results were expressed as Mean ± SEM. SA: sodium arsenite, AZA: azelaic acid

*** Significantly different from the control group at *P*-value<0.001.

** Significantly different from the control group at *P*-value<0.01.

### Significantly different from the SA group at *P*-value<0.001.

## Conclusion

This study indicates that SA increased ROS generation, pro-inflammatory cytokines, apoptotic cell death, and insulin secretion in the rat pancreatic islets. At the same time, AZA was able to correct these effects. This proved that AZA can restore the anti-oxidant capacity of the cells and normalize the expression of inflammatory cytokines, apoptotic events, and insulin secretion. AZA’s ability to modulate the metabolic toxicity of SA in pancreatic islets supports the idea that AZA can be a promising preventive measure for counteracting the deleterious effects of environmental toxicants in developing metabolic disorders such as diabetes. 

## Declaration

We have not used AI tools or technologies to prepare this manuscript.
